# Understanding mobility and sexual risk behaviour among women in fishing communities of Lake Victoria in East Africa: a qualitative study

**DOI:** 10.1186/s12889-020-09085-7

**Published:** 2020-06-15

**Authors:** Zachary Kwena, Sarah Nakamanya, Gertrude Nanyonjo, Elialilia Okello, Pat Fast, Ali Ssetaala, Bertha Oketch, Matt Price, Saidi Kapiga, Elizabeth Bukusi, Janet Seeley, the LVCHR, Heiner Grosskurth, Anatoli Kamali, Pontiano Kaleebu, Freddie Mukasa Kibengo, William Kidega, Jan De Bont

**Affiliations:** 1 Research Care and Training Program (RCTP), Kenya Medical Research Institute, Kisumu, Kenya; 2 Medical Research Council/Uganda Virus Research Institute MRC/UVRI and London School of Hygiene and Tropical Medicine (LSHTM) Uganda Research Unit, Entebbe, Uganda; 3 Uganda Virus Research Institute-International AIDS Vaccine Initiative (UVRI-IAVI) Project, Uganda Virus Research Institute, Entebbe, Uganda; 4 Mwanza Intervention Trials Unit (MITU), National Institute for Medical Research, Mwanza, Tanzania; 5 International AIDS Vaccine Initiative (IAVI), New York, USA; 6 London School of Hygiene and Tropical Medicine, London, UK

**Keywords:** Fishing communities, women’s mobility patterns, High HIV risk behaviour, Women’s livelihoods, Lake Victoria

## Abstract

**Background:**

HIV-prevalence and incidence is high in many fishing communities around Lake Victoria in East Africa. In these settings, mobility among women is high and may contribute to increased risk of HIV infection and poor access to effective prevention and treatment services. Understanding the nature and patterns of this mobility is important for the design of interventions. We conducted an exploratory study to understand the nature and patterns of women’s mobility to inform the design of HIV intervention trials in fishing communities of Lake Victoria.

**Methods:**

This was a cross-sectional formative qualitative study conducted in six purposively selected fishing communities in Kenya, Tanzania and Uganda. Potential participants were screened for eligibility on age (18+ years) and having stayed in the fishing community for more than 6 months. We collected data using introductory and focus group discussions, and in-depth interviews with key informants. Data focused on: history and patterns of mobility, migration in and out of fishing communities and the relationship between mobility and HIV infection. Since the interviews and discussions were not audio-recorded, detailed notes were taken and written up into full scripts for analysis. We conducted a thematic analysis using constant comparison analysis.

**Results:**

Participants reported that women in fishing communities were highly mobile for work-related activities. Overall, we categorized mobility as travels over long and short distances or periods depending on the kind of livelihood activity women were involved in. Participants reported that women often travelled to new places, away from familiar contacts and far from healthcare access. Some women were reported to engage in high risk sexual behaviour and disengaging from HIV care. However, participants reported that women often returned to the fishing communities they considered home, or followed a seasonal pattern of work, which would facilitate contact with service providers.

**Conclusion:**

Women exhibited circular and seasonal mobility patterns over varying distances and duration away from their home communities. These mobility patterns may limit women’s access to trial/health services and put them at risk of HIV-infection. Interventions should be tailored to take into account mobility patterns of seasonal work observed in this study.

## Background

HIV continues to be a public concern in many parts of the world especially in sub-Saharan Africa. By the end of 2018, an estimated 37.9 million people globally were living with HIV, while 1.7 becoming newly infected in the same year despite the progress in treatment. Sub-Saharan Africa is the most affected region accounting for a half of the people living with HIV [1].

Fishing communities of Lake Victoria in East Africa have high HIV prevalence and incidence, including among women resident and working in these communities [2–5]. In some age groups, more than half the women are living with HIV [6]. Women working in service outlets such as food kiosks, bars and recreational facilities are often the most affected. For instance, the proportion of women infected with HIV is twice as high as that of men of the same age [3, 4, 7]. HIV infection risk for women has been attributed to various socio-behavioural and economic vulnerabilities [8–11]. One of these vulnerabilities is the involvement in multiple concurrent sexual partnerships including fish-for-sex relationships (where fish are secured for or provided at favourable rates to women who provide sex). Fishermen follow fish migration patterns to secure good fish catch. Women fish traders, and even those involved in fish processing activities, such as scaling, drying, and smoking, have to follow the fish and the men. These women’s mobility patterns revolve around fish-landing beaches, retail markets and their rural homes [9, 12, 13].

In the context of high HIV prevalence and incidence, women’s mobility affects both HIV prevention and care [3, 14, 15]. For instance, mobility has been associated with exposure to involvement in high risk behaviour, especially in the fishing communities where fish-for-sex relationships occur. Understanding mobility requires that its nature (i.e. inherent features and characteristics of travel) as well as patterns (i.e. route, course and timings of travels) are described [16]. In the course of their work, women may travel to unfamiliar places and develop relationships with men they do not know well to get priority in the sale of fish [12]. Additionally, mobility is known to interrupt treatment schedules, affecting retention in care for women enrolled in HIV programs [14]. Given women’s susceptibility to HIV infection in these settings [7], developing and testing targeted interventions tailored for them is critically important as we move towards UNAIDS goal of AIDS elimination by 2030 [17, 18].

Researchers have interest in populations at high risk of HIV infection, such as women in the fishing communities. This is not only because scholars want to contribute to testing interventions that prevent further infections but also because this is a population that can support achieving HIV study endpoints [19]. Successful testing of interventions requires regular contact with the women for follow up and other study-related procedures [20–23]. While women working in fishing communities on Lake Victoria are known to be highly mobile, with risks of HIV infection and loss from HIV care [4], details of the nature and patterns of their mobility are scant. Thus, we sought to document the nature and patterns of mobility of women in six fishing communities on Lake Victoria, with a view to using the data to develop a larger HIV intervention study.

## Methods

### Study design

This was a cross-sectional formative qualitative study to gather information on nature and patterns of women’s mobility required in planning future intervention studies in fishing communities. The study was conducted between February and June 2018 among fishing communities of Lake Victoria in Kenya, Uganda and Tanzania.

### Study settings

This study was conducted in six purposively selected fishing communities (with a known HIV prevalence of > 15% among women and a population of at least 1000 people) on the shores of Lake Victoria in the three East African countries of Kenya, Uganda and Tanzania. Two communities were selected from each country. Within the context of this study, a fishing community consisted of one or more landing sites and on both mainland and island where people were focused on fishing and fishing-related activities, and lived together in a defined geographical area. In some cases fishing communities coincided with administrative boundaries such as villages and wards. Landing sites in these fishing communities are often very busy, attracting a large number of people who come to engage in fish-related activities. As a result, there is often intense competition for traders to access fish for markets. Overall, fishing community residents are known to exhibit high HIV risk behaviour and have corresponding high prevalence and incidence. Although our study population comprised women resident and/or working in fishing communities, we collected data from both women as well as from men about women in their communities.

### Sampling procedure

The process of identifying participants started by conducting community entry visits to introduce the study and the research team to community gatekeepers as well as obtaining community buy-in. Beach management officials and local council leaders who are the custodians of the landing sites provided logistical support. The logistical support mostly included finding meeting venues and assisting in identifying potential participants with required characteristics. The study team approached potential participants and screened them for eligibility that included age above 18 and at least 6 months duration in the community. The participants were then scheduled for interviews or group discussions at mutually agreed venue and time. Some of these participants were identified during community entry meetings while others were referrals from participants who had participated in previous study activities and knew people who were knowledgeable on the issues we were collecting information on.

### Consenting and data collection

The study protocol was approved for implementation by KEMRI’s Scientific and Ethics Review Unit in Kenya (SERU#3593), Uganda Virus Research Institute Ethics Committee and National Council for Science and Technology in Uganda (UVRI REC#605 and SS#4470) and, the National Health Research Ethical Committee (NatHREC) (NIMR#2654) in Tanzania. All identified participants were given details about the study and all provided written informed consent before they were recruited in the study. We did not audio-record the discussions but instead took detailed notes and expanded them soon after the interview/discussion. This is because we wanted, to the extent possible, to collect data from participants while going about their usual business such as working or resting after their day’s activities. Also, there have been concerns about audio-recording in some settings that diminish the quality of data collected [24]. This approach allowed us to ensure that the participants had discussions in a natural setting devoid of fears often associated with a recorder.

We collected data using introductory group sessions, focus group discussions and in-depth interviews (see Table 1). The data collected focused on: history of the landing sites, migrations in and out of the communities, mobility and related factors, role of women at the landing sites, women’s mobility patterns and, relationship between mobility and HIV infections. The focus group discussion and in-depth interview guides were developed in English and translated into the local languages of Kiswahili, Dholuo and Luganda. On average, the group discussions took approximately 60 min while in-depth interviews lasted for between 30 and 45 min.

**Table 1:** Number of participants recruited in each country by gender

	Kenya		Tanzania		Uganda	
	Male	Female	Male	Female	Male	Female
Introductory group meeting	9	10	8	10	15	14
Community group discussions						
Group 18–25 years	9	35	18	24	22	12
Group 25+ years	11	32	16	20	22	13
In-depth interviews	4	6	6	14	10	10

We conducted a total of six introductory group sessions one in each of the six fishing communities. Each session consisted of 8–15 key informants who had lived in the fishing communities for at least 1 year and were expected to have adequate local knowledge. The key informants who participated in the introductory sessions were nominated by local leaders in the communities. The introductory group sessions provided data that helped to determine the perceptions of men and women on women’s livelihoods as well as the role of mobility in women’s lives. We conducted 24 community group discussions on the perceptions of men and women on livelihoods and the role of mobility in women’s lives. Group discussion participants were identified in two ways: (1) selected by community local leaders and, (2) referrals from introductory group session participants of people who were knowledgeable on the subject of discussion. The groups were divided into the following four categories based gender and age: young women (18–25), older women (25+), young men (18–25) and older men (25+). Unlike the introductory group sessions, each focus group discussion comprised slightly fewer participants (8–12). We also conducted 50 individual in-depth interviews. To obtain the 50 individuals interviewed, we identified at least six individuals in each of the six participating fishing communities. We approached the identified individuals for possible participation until we had recruited 50 into the study. The 50 participants recruited included: beach management unit officials, village health teams and elders and women.

### Data management and analysis

The notes taken during the focus group discussions and in-depth interviews were written out into expanded scripts and saved in password-protected files on study computers. The files were also saved in external hard drives and kept offsite for security reasons. We started analysis by scanning through the data to develop broad codes and eventually fine codes. To ensure that the resultant coding reports from the four teams were harmonized, we held audio meetings to refine the coding framework and approve a codebook that was used across the teams. The broad codes represented the main thematic areas that included: (a) history of women’s mobility in fishing communities, (b) mobility and women’s livelihoods, (c) women’s mobility patterns and, (d) association between mobility and HIV. Women’s mobility was categorized as over long distances traveling across county/district or international boundaries and short distances traveling within county/district boundaries. Analysis was guided by the topics we used to develop the discussion and interview guides. We also incorporated data-led analysis where analytical themes were allowed to emerge from the scripts to define fine codes during the process of reading, exploration and coding responses [25]. Four teams manually coded the scripts and shared the coding reports. We used constant comparison to ensure consistency in coding across the sites. Through regular conference calls with teams from different sites, we were able to discover dominant salient processes and the participants’ perspectives across the sites.

## Results

### History of women’s mobility in fishing communities

Participants gave historical accounts of population dynamics in the fishing communities; mostly resulting from migration. Initially, men established the fishing sites and were later joined by women to help them with domestic chores such as cooking. Young people in Kenya, for example, mentioned this happening in the time of their grandparents. A participant in Uganda explained the process:

*Before, this place had very few women. These [women] came with men that came to fish. They were brought to cook for them and some of these came with their husbands…Every time they moved, they moved with everything that belonged to them from place to place looking for places with good fish catches (UG, FGD older woman).*

This population growth also increased the demand for services such as guest houses, bars and food kiosks, boat building and repairs as well as shops selling different merchandise. High demand for fish in urban establishments triggered mobility, especially among women. The women made circular trips between fish communities to buy fish, markets to sell their fish and rural hinterlands to visit families, seek health services and attend to their crops. Additionally, women who had separated from their spouses or were widowed migrated to access work or business opportunities at fish-landing sites that required relatively small capital to begin. As the numbers of these women increased, local communities begun associating negative behaviours that were happening with migrant populations, especially widowed or divorced women as illustrated in the quote below:

*It is rare to find girls born in this community in such groups [sex work], the majority of women involved in this business are migrants from other areas. Our community has so many migrants. These young women are usually on transit to major islands. But sometimes they can stay around for a few days or weeks or even months depending on the fish season which is the main determinant of “business opportunities” (TZ, FGD older man).*

### Mobility and women’s livelihoods

Although women in the fishing communities were mainly involved in fishing-related business, they were not a homogenous group. There were women with comparatively large capital investments who targeted markets in major urban centers and even other countries. Women with modest capital focused on buying fish from fishermen and selling in local nearby markets. To collect enough fish for market, some women, especially those dealing with dried fish, camped at the fish-landing beaches for several days or moved from beach to beach to collect enough fish before taking it to the markets. There were other women solely hired at the beaches to undertake various fish processing activities.

*“Women are involved in different activities and work. Many come to buy fish, sorting, scaling, dissecting, washing and drying fish. All these activities are done either for income or consumption...” (KE, IDI- woman, 53 years)*.

Other women were reported to be involved in the service industry, running small businesses such as food kiosks, bars and shops as well as groceries selling vegetables, fruits. In some cases, these women were the main employers of young women arriving in the fishing communities. Participants noted that the work options available for women, especially new arrivals into the community, were mostly low-paying jobs like bar/restaurant work and sex work.

*“Number one job is bar work. They do not do this job for a pay but stage here as a waiting place for potential sexual clients. In addition to this, a woman could as well earn extra money from men who offer her drinks. ‘If he buys me a drink, I instead fill the beer bottle with water and take that and keep the money. I send this money which I have ‘swindled’ to the caretaker of my children”. (UG, FGD Young woman).*

Community members had mixed perceptions about women’s mobility. Women with established businesses were viewed as respectable and worked to provide for their families. However, others like those who worked in bars and restaurants were described as a problem because of their sexual behaviour:

“*Majority of these transaction sex workers are marriage breakers. There were, for example, three ladies whose photos were put on the village notice board, showing that they had been chased out of this community and barred from returning to the community” (TZ, IDI-woman, aged 47).*

Participants observed that most of the work that involved intense travelling was done by single women who worked as sex workers and/or in restaurants and bars. They were free to move from one beach to another based on fish catch and flow of money.

*“Sex workers are the most mobile. They may come to the place and stay for like a week and when they are still new, they get many sexual clients but as the men get used to them, clients reduce and that is when they move to another fishing community and continue in that kind of circle” (UG, FGD Young woman).*

Other women, including some who were married, had migrated to fishing communities with a hope of raising capital to start their own businesses. Initially, such women may start working in establishments like bars, salons and restaurants but may also engage in sex for money to raise their initial capital.

*“There is something that we call sex for fish. Some women come without any capital and they want to deal in fish. So, she gets in touch with someone who owns a boat. So, with time as she fails to get the money to pay, she ends up paying in kind and the man keeps providing her with fish” (UG, Introductory group session, gatekeepers).*

### Women’s mobility patterns

Women often travelled in search of opportunities to earn an income. However, they also travelled for other reasons such as joining their families, seeking medical care, avoiding arrest (if involved in illegal activities), and for leisure or separation due to domestic violence.

*Many people do not have capital to begin trading fish. They keep moving from one beach to another to try their luck. They move for leisure, in search of work, to get married, fishing and trading in fish.” (KE, young man < 25 years*).

The amount of time women stayed away ranged from a couple of days to several months. The type of work or business the women were involved in determined the timing. Women involved in fish-related activities and those directly involved in sex work had more complicated mobility patterns than women involved in other activities such as food vending, bar/lodge businesses. Women also moved when the fishermen moved.

*“Work at the landing site hinges on the lake. If there is fish, most of the people will stay around and if they hear there is too much fish in xxx, all the boats will head there. Some fishermen also have women here so when they go away, their women also go away”. (UG, Introductory group session, gatekeepers).*

Long distance mobility was common among women involved in bulk buying and selling and targeting markets in far off locations to sell their fish. For instance, in Tanzania, these women would buy their fish in Mwanza and travel to sell them as far as Arusha (about 680 km), Shinyanga (about 180 km) and Geita (about 140 km). This group of women were mostly described as older women and with higher income. They travelled back and forth between fishing communities and their market destinations; often spending up to 2 weeks away each trip and travelling at least once every month. The mobility of this group was mainly determined by the availability of fish and fish market opportunities.

Short distance mobility was common among women engaged in medium scale fish business and those involved in non-fish related business such as charcoal and firewood buying and selling. This group travelled between fishing communities and surrounding towns and rural areas at least once a month, sometimes staying out of the community for up to 7 days per trip. Women who were involved in charcoal and firewood business were mostly older compared to those who were engaged in fish businesses.

In some communities, women employed in fishermen’s camps tended to be less mobile than those involved in fish trading. This is because they worked at premises owned by boat owners where fishermen returning from fishing expeditions take a bath, change clothes, find food and rest. These camps are managed by women over the age of 35 years, who in turn recruit younger women, usually aged between 18 and 25 years to help them with cooking and other activities. Women employed in such settings are less mobile because they are employed on contract, sometimes up to 6 months’ duration. These women moved only if the fishing crew that they care for are relocated to another fishing community or island, or if their contract expired and they had to find other jobs. 

As noted above, frequent mobility was mainly among women involved in sex work or transactional sex and working in bars and restaurants.

“*[the] majority of us women working in bars move from one place to another looking for places with good business. It does not matter where this money is, it may be in a fishing community, an island or a mining area. We will usually go wherever there is good business returning to xxx only when it gets busy again. Sometimes it can take up to 3 months before we return to this fishing community (TZ, IDI female bar attendant).*

In Tanzania, women who worked in bars and also provided sexual services are known in the community as “women who hunt for fish heads”, likening sex clients to fish heads, a part of fish considered ‘juicier’ by fish eaters. Based on their complex mobility patterns that involve moving between their areas of origin, fishing communities and nearby cities and major towns, they are described as freelance sex workers. A distinctive characteristic of women involved in selling sex was that they were able to pay rent, eat and dress well although they did not seem to have recognizable livelihood activities.

“*… .and other women here have no job completely but you can see them they get money, they eat and drink* also, *women here exchange sex for material. Example: a man can give a woman fish and be given sex. This has led to moral and marriages destruction at a great extent … only few women here have self-respect” (TZ, IDI-woman aged 55).*

In addition to the reasons for mobility outlined above, seasonal weather changes like the strong winds that normally blow from late July to October, heavy rains that cause flooding as well as the changing fish migration patterns, reportedly influenced the nature of mobility. Movements arising from the natural phenomena were mostly long-term as people moved away from the community for several months until the season changed or the danger passed. For instance, during the full moon when the silver fish (*omena*) catch is low, fishermen and women traders who deal in this fish species use the time to travel to their rural homes to see their families and attend to their crops.

### Women’s mobility, high risk behaviour and access to healthcare services

Participants linked mobility with the risk of HIV infection in the fishing communities. They specifically blamed women migrating into the fishing communities for bringing HIV into their sites:

*Some women come here when they are already infected with HIV. When the fishermen see them, they get attracted and begin to relate with them especially the jaboya (fish for sex). You will find that even when there is no fish, the jaboya will make sure that these specific women get fish and, in the process, HIV spreads* (KE, IDI woman aged 53).

Participants explained that people take advantage of being away from their communities of origin to engage in high risk behaviour. This is because they are usually not well-known at their destinations and therefore nobody can hold them socially accountable.

*There is a big relationship between mobility and HIV risk; “sex for fish” that leads to high rates of HIV infection. The women looking for fish to buy must first accept to have sex with the boat owners or the agents. Men who move out from xxx for fishing have sex with women because their wives are not there with them. (KE IDI Woman aged 25).*

Women in business/traders, especially the hawkers were reported to have different sexual partners at every stopover or place where they sell their merchandise. This is to ensure that there was always somebody to foot their bills at every stopover during their movements.

Unfortunately, condom use among the mobile women was generally limited. Participants observed that even women who initially insist on condoms relax this requirement after sometime with regular partners. This is because of familiarity with their partner. As illustrated in the quote below, this has potential to spread infections to families back at home.

“… *Yet when a man comes to befriend you and you agree to have a relationship, he could decide to use a condom for a month, after that, he would gain confidence that I’m not infected and he decides to do away with the condom. In that case if I’m HIV positive, he has picked it from me and will take it back to the wife at home”*. *(UG, FGD, older women).*

Participants reported that antiretroviral treatment for women living with HIV improves their health to the extent that it becomes impossible to even imagine that they may be infected. Such women attract men when they travel from communities where they are known to places where they are strangers.

*ARVs uptake is making people to be fat and beautiful. Someone [living with HIV] may move from xxx to xxx and come back through xxx while interacting with several men sexually through the entire trip. This shows how movement can cause HIV infection. (KE, FGD younger men).*

Participants also linked women’s mobility with missing taking ARVs and defaulting their treatment altogether. This was especially so for those who travelled over long distance and spent significant amount of time from home.

*Some women are on ARVs to be taken at designated time and because of their movements, they sometime default when they go away from their clinics (KE, FGD older men)*.

## Discussion

This study set out to assess the nature and patterns of women’s mobility and its perceived relationship with high risk sexual behaviour and HIV infection in the fishing communities. We found that women in the fishing communities are highly mobile and their mobility dates back to the establishment of fish-landing sites when they joined men to support them with household chores. Women ran their own fish and other small businesses or worked as attendants in food kiosks, bars and restaurants. We also found that most women were engaged in livelihood activities that required them to be mobile in search of income generating opportunities to support their families. We categorized women mobility in the fishing communities over long and short distances or different time periods depending on the kind of business they were involved. Mobility takes women outside their social network and healthcare access boundaries which makes them susceptible to engaging in high risk sexual behaviour and disengaging from care for those enrolled in HIV care.

Similar to findings of other migration and mobility studies elsewhere [26, 27], women’s mobility in the fishing communities is mainly driven by the search for economic opportunities to support their families [28]. Mobile individuals make decisions to travel from their origin to areas perceived to have more opportunities with hopes of finding employment or engaging in business activities for survival. Indeed, studies have shown that those who end up moving often experience profound change of livelihoods and improve their socio-economic situation at their destinations [27]. For instance, Pearson and colleagues observe that women who arrive in fishing communities with ready capital to establish their own businesses are able to avoid lower paid and riskier work like fishing processing that expose them to temptations of establishing sexual relationships with fishermen [28].

Apart from women who migrate in search of employment/business opportunities, there are those whose work or business requires them to be mobile. For instance, women fish traders make circular movements between fishing beaches, markets and their rural homes sometimes over long periods [13]. Such women could easily be traced for research/health interventions at their destinations or when they returned to the fishing communities they considered as their homes. Providing interventions that target these women requires sound understanding of their mobility patterns so that researchers that enrol them in clinical trials and even healthcare providers are clear on when, how and where to access the women for the follow up visits or their clinic appointments.

As important as mobility may be to an individual’s economic survival, it has also been associated with interrupting lifestyles, access to healthcare services and elevating risk profiles of those affected [14, 29, 30]. In this study we found that mobility was associated with high risk sexual behaviour that exposed individuals to HIV infections. This has been corroborated by other studies [31–33]. For instance, in a study of the effect of mobility on sexual behaviour among MSM in India, Ramesh and colleagues found that higher proportion of men who were mobile reported unprotected sex and were also likely to be HIV infected [31]. However, Colebunders and colleagues note that a high HIV prevalence in a mobile population does not necessarily mean that mobility itself increases susceptibility to HIV but rather behaviours of mobile individuals outside their social audit boundaries may be responsible for the high prevalence [34].

Testing HIV prevention and treatment interventions in HIV hotspots such as fishing communities [3, 7, 35] require fairly stable and accessible populations for follow up visits. Mobility, especially away from local communities and for long periods can severely jeopardize retention that is crucial in monitoring and addressing untoward events and providing additional support services as may be required. Similarly, many studies have associated mobility with poor adherence to ARVs and retention in HIV care leading to treatment interruption and poor health outcomes [14, 36–38]. Indeed, HIV care programs are experiencing challenges with patient retention [39–42]. Recent studies show that over one quarter of patients enrolled in HIV care programs in sub-Saharan Africa are lost to follow up at 12 months post ART initiation [43] and, mobility that takes patients away from their HIV care clinics is listed as one of the reasons [14].

We observe a few limitations in this study. One, although we collected data from a number of sites along Lake Victoria, the study was not designed in a way to allow for direct comparison between sites to establish if there exist discernible differences in women’s mobility patterns. Also, these sites were not sampled in a way to give representative results for all fishing communities in the 3 countries. Two, this being a cross-sectional formative qualitative study, application of its findings is limited to local fishing communities where data was collected; additionally, no causation can be inferred between variables. Despite the limitations, this study provides crucial in-depth data on women’s mobility patterns that can not only inform the design of intervention studies in the fishing communities but also provision of HIV prevention and treatment health services to the women. Consequently, adopting and scaling up interventions resulting from such trials in communities with high HIV incidence significantly contributes towards reducing new infections as we approach elimination of HIV by 2030.

## Conclusion

In conclusion, women’s history in the fishing communities is old as the establishment of the fish landing sites. Women at these fishing communities are involved in livelihood activities that require them to have circular movements over long and short distances and periods. Women’s travel beyond their boundaries of social audit and health care makes them vulnerable to high risk sexual behaviour and poor retention in care and other intervention services. Any clinical trials, epidemiological studies, or preventive or care interventions targeting these women will need to incorporate the timings of their circular movements between the fishing communities, urban fish markets and their rural hinterlands.

## Supplementary information

**Additional file 1.**

## Data Availability

The datasets used and/or analysed during the current study are available from the corresponding author on reasonable request.

## Abbreviations

AIDS: Acquired Immunodeficiency Syndrome
ART: Anti-retroviral Therapy
ARV: Anti-retroviral
FGD: Focus Group Discussion
HIV: Human Immunodeficiency Virus
IAVI: International AIDS Vaccine Initiative
IDI: In-depth Interview
KE: Kenya
KEMRI: Kenya Medical Research Institute
LSHTM: London School of Health and Tropical Medicine
LVCHR: Lake Victoria Consortium for Health Research
MITU: Mwanza Intervention Trials Unit
MRC: Medical Research Council
MSM: Men who Have sex with Men
NatHREC: National Health Research Ethical Committee
RCTP: Research Care and Training Program
SERU: Science and Ethics Review Unit
TZ: Tanzania
UG: Uganda
UK: United Kingdom
UNAIDS: Joint United Nations Program on HIV and AIDS
USA: United States of America
UVRI: Uganda Virus Research Institute

## References

[1] UNAIDS (2019). Fact sheet - World AIDS day 2019.

[2] Asiki G, Mpendo J, Abaasa A, Agaba C, Nanvubya A, Nielsen L (2011). HIV and syphilis prevalence and associated risk factors among fishing communities of Lake Victoria, Uganda. Sex Transm Infect.

[3] Seeley J, Nakiyingi-Miiro J, Kamali A, Mpendo J, Asiki G, Abaasa A (2012). High HIV incidence and socio-behavioral risk patterns in fishing communities on the shores of Lake Victoria, Uganda. Sex Transm Dis.

[4] Kwena ZA, Camlin CS, Shisanya CA, Mwanzo I, Bukusi EA (2013). Short-term mobility and the risk of HIV infection among married couples in the fishing communities along Lake Victoria, Kenya. PloS One.

[5] Abaasa A, Asiki G, Price MA, Ruzagira E, Kibengo F, Bahemuka U (2016). Comparison of HIV incidence estimated in clinical trial and observational cohort settings in a high risk fishing population in Uganda: implications for sample size estimates. Vaccine..

[6] Chang LW, Grabowski MK, Ssekubugu R, Nalugoda F, Kigozi G, Nantume B (2016). Heterogeneity of the HIV epidemic in agrarian, trading, and fishing communities in Rakai, Uganda: an observational epidemiological study. Lancet HIV..

[7] Kwena ZA, Njuguna SW, Ssetala A, Seeley J, Nielsen L, De Bont J (2019). HIV prevalence, spatial distribution and risk factors for HIV infection in the Kenyan fishing communities of Lake Victoria. PLoS One.

[8] Kwena ZA, Shisanya CA, Bukusi EA, Turan JM, Dworkin SL, Rota GA (2017). Jaboya (‘sex for fish’): a qualitative analysis of contextual risk factors for extramarital partnerships in the fishing communities in Western Kenya. Arch Sex Behav.

[9] Kwena Z, Mwanzo I, Shisanya C, Camlin C, Turan J, Achiro L (2014). Predictors of extra-marital partnerships among women married to fishermen along Lake Victoria in Kisumu County. Kenya PloS One.

[10] Kiene SM, Sileo KM, Dove M, Kintu M (2019). Hazardous alcohol consumption and alcohol-related problems are associated with unknown and HIV-positive status in fishing communities in Uganda. AIDS Care.

[11] Kiwanuka N, Ssetaala A, Ssekandi I, Nalutaaya A, Kitandwe PK, Ssempiira J (2017). Population attributable fraction of incident HIV infections associated with alcohol consumption in fishing communities around Lake Victoria, Uganda. PloS One.

[12] Camlin CS, Kwena ZA, Dworkin SL (2013). Jaboya vs. jakambi: status, negotiation, and HIV risks among female migrants in the ‘sex for fish’ economy in Nyanza Province, Kenya. AIDS Educ Prev Off Publ Int Soc AIDS Educ.

[13] Camlin CS, Kwena ZA, Dworkin SL, Cohen CR, Bukusi EA (2014). ‘She mixes her business’: HIV transmission and acquisition risks among female migrants in western Kenya. Soc Sci Med 1982.

[14] Camlin CS, Cassels S, Seeley J (2018). Bringing population mobility into focus to achieve HIV prevention goals. J Int AIDS Soc.

[15] Clouse K, Fox MP, Mongwenyana C, Motlhatlhedi M, Buthelezi S, Bokaba D (2018). ‘I will leave the baby with my mother’: Long-distance travel and follow-up care among HIV-positive pregnant and postpartum women in South Africa. J Int AIDS Soc.

[16] Panigutti C, Tizzoni M, Bajardi P, Smoreda Z, Colizza V (2017). Assessing the use of mobile phone data to describe recurrent mobility patterns in spatial epidemic models. R Soc Open Sci.

[17] Krebs E, Min JE, Bayoumi AM, Barrios R, Montaner JSG, Nosyk B (2018). Informing targeted interventions to optimize the cascade of HIV care using cluster analyses of health resource use among people living with HIV/AIDS. AIDS Behav.

[18] Kumar R, Mehendale SM, Panda S, Venkatesh S, Lakshmi P, Kaur M (2011). Impact of targeted interventions on heterosexual transmission of HIV in India. BMC Public Health.

[19] Ruzagira E, Wandiembe S, Abaasa A, Levin J, Bwanika A, Bahemuka U (2011). Prevalence and incidence of HIV in a rural community-based HIV vaccine preparedness cohort in Masaka, Uganda. PloS One.

[20] Kiwanuka N, Mpendo J, Asiimwe S, Ssempiira J, Nalutaaya A, Nambuusi B (2018). A randomized trial to assess retention rates using mobile phone reminders versus physical contact tracing in a potential HIV vaccine efficacy population of fishing communities around Lake Victoria. Uganda BMC Infect Dis.

[21] Babu GR, Karthik M, Ravi D, Ana Y, Shriyan P, Hasige KK (2018). What makes the pregnant women revisit public hospitals for research? Participant engagement and retention trial in a public hospital (PERTH): an RCT protocol. BMC Pregnancy Childbirth.

[22] Wynne J, Muwawu R, Mubiru MC, Kamira B, Kemigisha D, Nakyanzi T (2018). Maximizing participant retention in a phase 2B HIV prevention trial in Kampala, Uganda: the MTN-003 (VOICE) study. HIV Clin Trials.

[23] Abaasa A, Asiki G, Mpendo J, Levin J, Seeley J, Nielsen L (2015). Factors associated with dropout in a long term observational cohort of fishing communities around Lake Victoria, Uganda. BMC Res Notes.

[24] Rutakumwa R, Mugisha JO, Bernays S, Kabunga E, Tumwekwase G, Mbonye M (2019). Conducting in-depth interviews with and without voice recorders: a comparative analysis. Qual Res.

[25] Mack N, Woodsong C, MacQueen K, Guest G, Namey E. Qualitative research methods: a data collector’s field guide: Family Health International; 2005. p. 118.

[26] van Geel J, Valentina M (2018). Conceptualising youth mobility trajectories: thinking beyond conventional categories. J Ethnic Migr Stud.

[27] Hoffmann EM, Konerding V, Nautiyal S, Buerkert A (2019). Is the push-pull paradigm useful to explain rural-urban migration? A case study in Uttarakhand, India. PloS One.

[28] Pearson G, Barratt C, Seeley J, Ssetaala A, Nabbagala G, Asiki G (2013). Making a livelihood at the fish-landing site: exploring the pursuit of economic independence amongst Ugandan women. J East Afr Stud J Br Inst East Afr.

[29] Wesolowski A, O’Meara WP, Eagle N, Tatem AJ, Buckee CO (2015). Evaluating spatial interaction models for regional mobility in sub-Saharan Africa. PLoS Comput Biol.

[30] Deane KD, Samwell Ngalya P, Boniface L, Bulugu G, Urassa M (2018). Exploring the relationship between population mobility and HIV risk: evidence from Tanzania. Glob Public Health.

[31] Ramesh S, Mehrotra P, Mahapatra B, Ganju D, Nagarajan K, Saggurti N (2014). The effect of mobility on sexual risk behaviour and HIV infection: a cross-sectional study of men who have sex with men in southern India. Sex Transm Infect.

[32] Saggurti N, Schensul SL, Verma RK (2009). Migration, mobility and sexual risk behavior in Mumbai, India: Mobile men with non-residential wife show increased risk. AIDS Behav.

[33] Kishamawe C, Vissers DCJ, Urassa M, Isingo R, Mwaluko G, Borsboom GJJM (2006). Mobility and HIV in Tanzanian couples: both mobile persons and their partners show increased risk. AIDS Lond Engl.

[34] Colebunders R, Kenyon C (2015). Behaviour, not mobility, is a risk factor for HIV. Lancet HIV.

[35] Mafigiri R, Matovu JKB, Makumbi FE, Ndyanabo A, Nabukalu D, Sakor M (2017). HIV prevalence and uptake of HIV/AIDS services among youths (15–24 Years) in fishing and neighboring communities of Kasensero, Rakai District, South Western Uganda. BMC Public Health.

[36] Phillips TK, Clouse K, Zerbe A, Orrell C, Abrams EJ, Myer L (2018). Linkage to care, mobility and retention of HIV-positive postpartum women in antiretroviral therapy services in South Africa. J Int AIDS Soc.

[37] Clouse K, Vermund SH, Maskew M, Lurie MN, MacLeod W, Malete G (2017). Mobility and Clinic Switching Among Postpartum Women Considered Lost to HIV Care in South Africa. J Acquir Immune Defic Syndr 1999.

[38] Mukumbang FC, Mwale JC, van Wyk B (2017). Conceptualising the factors affecting retention in care of patients on antiretroviral treatment in Kabwe District, Zambia, using the ecological framework. AIDS Res Treat.

[39] Mee P, Rice B, Lemsalu L, Hargreaves J, Sambu V, Harklerode R, et al. Changes in patterns of retention in HIV care and antiretroviral treatment in Tanzania between 2008 and 2016: an analysis of routinely collected national programme data. J Glob Health. 2019;9(1) Available from: http://jogh.org/documents/issue201901/jogh-09-010424.pdf. [cited 2019 May 15]. 10.7189/jogh.09.010424 PMC6445500 30992984

[40] Bulsara SM, Wainberg ML, Newton-John TRO (2018). Predictors of adult retention in HIV care: a systematic review. AIDS Behav.

[41] Roy M, Czaicki N, Holmes C, Chavan S, Tsitsi A, Odeny T (2016). Understanding sustained retention in HIV/AIDS care and treatment: a synthetic review. Curr HIV/AIDS Rep.

[42] Geng EH, Nash D, Kambugu A, Zhang Y, Braitstein P, Christopoulos KA (2010). Retention in care among HIV-infected patients in resource-limited settings: emerging insights and new directions. Curr HIV/AIDS Rep..

[43] Haas AD, Tenthani L, Msukwa MT, Tal K, Jahn A, Gadabu OJ (2016). Retention in care during the first 3 years of antiretroviral therapy for women in Malawi’s option B+ programme: an observational cohort study. Lancet HIV..

