# Supplementary material for: Understanding mobility and sexual risk behaviour among women in fishing communities of Lake Victoria in East Africa: a qualitative study
Source: BMC Public Health. 2020 Jun 15;20:944. doi: 10.1186/s12889-020-09085-7 (PMC7296721; doi:10.1186/s12889-020-09085-7)
Supplement: Supplementary file 1 — Additional file 1. [file 12889_2020_9085_MOESM1_ESM.doc]

# MOBILITY PATTERNS AND FEASIBILITY OF TRACKING WOMEN AT HIGH RISK OF HIV IN THE FISHING COMMUNITIES IN KENYA, TANZANIA AND UGANDA

# IN-DEPTH INTERVIEW GUIDE

1. How was this beach established?
   1. Probe for community/beach leadership, population and type of people doing fishing within the community/beach
   2. What is the proportion of men, women and children?
   3. Describe to me the proportion of married and unmarried people in this community
2. Where are people on this beach come from? Neighbouring communities? Other communities within the location, county?
3. Describe to me the movement of people between this beach and elsewhere? When do they move and how for how long?
4. Is there difference in mobility between men and women, young and old? How different are they?
5. What are the factors/reasons for movement in and out of this beach? What are some of the challenges women and other people face in this community when they move in and out?
6. What activities/work are women involved in? Has these activities/work changed over time? What did women do in the past to make a living? Is that different now?
7. What do you think is the relationship between mobility and the risk of HIV infection? How do you think people are exposed when they travel?
8. What do people say about ‘HIV risk’ and mobility?
   1. Probe to find out if they are linked in people’s minds or not.
   2. Probe for opinions on women moving for work, for marriage, for other reasons
9. If we wanted to track women's mobility to understand their pattern of movement and the places they go to and how long they stay so that we can track them when we begin a longitudinal follow up study, how do you think we can track them?
10. How will women and other people in general feel about our research team tracking their (women's) movements?
11. If we gave women a small device which tracks movements to carry, how will they (women) and other people feel about it?
12. What will be the obstacles in carrying the device with them?
13. How do we tackle these obstacles?

# MOBILITY PATTERNS AND FEASIBILITY OF TRACKING WOMEN AT HIGH RISK OF HIV IN THE FISHING COMMUNITIES IN KENYA, TANZANIA AND UGANDA

# INTRODUCTION/COMMUNITY GROUP DISCUSSION GUIDE

1. How was this beach established?
   1. Probe for community/beach leadership, population and type of people doing fishing within the community/beach
   2. What is the proportion of men, women and children?
   3. Describe to me the proportion of married and unmarried people in this community
2. Where are people on this beach come from? Neighbouring communities? Other communities within the location, county?
3. Describe to me the movement of people between this beach and elsewhere? When do they move and how for how long?
4. Is there difference in mobility between men and women, young and old? How different are they?
5. What are the factors/reasons for movement in and out of this beach? What are some of the challenges women and other people face in this community when they move in and out?
6. What activities/work are women involved in? Has these activities/work changed over time? What did women do in the past to make a living? Is that different now?
7. What do you think is the relationship between mobility and the risk of HIV infection? How do you think people are exposed when they travel?
8. What do people say about ‘HIV risk’ and mobility?
   1. Probe to find out if they are linked in people’s minds or not.
   2. Probe for opinions on women moving for work, for marriage, for other reasons
9. If we wanted to track women's mobility to understand their pattern of movement and the places they go to and how long they stay so that we can track them when we begin a longitudinal follow up study, how do you think we can track them?
10. How will women and other people in general feel about our research team tracking their (women's) movements?
11. If we gave women a small device which tracks movements to carry, how will they (women) and other people feel about it?
12. What will be the obstacles in carrying the device with them?
13. How do we tackle these obstacles?
